# Synergistic function of RNA modifications in *Arabidopsis* and rice

**DOI:** 10.1007/s42994-025-00248-x

**Published:** 2025-10-09

**Authors:** Ancheng Ma, Shuaibin Wang, Xinxi He, Yongbo Qu, Shenglin Xie, Junping Gao, Yu Peng, Lisha Shen, Wenxuan Pu, Chongsheng He

**Affiliations:** 1 Tobacco Research Institute of Technology Centre, China Tobacco Hunan Industrial Corporation, Changsha, 410014 China; 2 Hunan Key Laboratory of Plant Functional Genomics and Developmental Regulation, College of Biology, Hunan University, Changsha, 410082 China; 3 Hunan Research Center of the Basic Discipline for Cell Signaling, Changsha, 410082 China; 4 Temasek Life Sciences Laboratory, National University of Singapore, Singapore, 117604 Singapore

**Keywords:** *Arabidopsis thaliana*, Rice, ac^4^C, m^6^A, m^5^C

## Abstract

The epigenomic landscape regulates gene expression and chromatin dynamics, with histone and RNA modifications playing crucial roles. Although studies have elucidated the interactions among chromatin modifications, DNA methylation, and mRNA modifications, the relationships among RNA modifications and their collective influence on RNA metabolism remain poorly understood. Grasping these epigenetic mechanisms is essential for improving crop resilience and productivity. In this study, we explored the co-occurrence and functional interactions of three significant mRNA modifications in Arabidopsis (*Arabidopsis thaliana*) and rice (*Oryza sativa*): *N*^*4*^-acetylcytidine (ac^4^C), *N*^*6*^-methyladenosine (m^6^A), and 5-methylcytosine (m^5^C). Our results indicate that these modifications frequently coexist in the same transcripts, exhibiting distinct spatial distributions across species. Notably, the m^6^A modification enhances the ac^4^C-mediated destabilization of RNA secondary structures, especially when modifications are clustered, thereby promoting RNA stability. In Arabidopsis, the ac^4^C modification improved translational efficiency and the m^6^A modification amplified this effect in a distance-dependent manner; by contrast, in rice the influence of m^6^A is independent of distance. The m^5^C modification has minimal impact on RNA structure or stability but modulates m^6^A-associated transcript stability in a context-dependent manner. Our findings shed light on the dynamic regulatory code of combinatorial RNA modifications, highlighting species-specific mechanisms of post-transcriptional regulation. This research offers valuable insights into the intricate interplay of RNA modifications, with implications for advancing agricultural biotechnology through a deeper understanding of plant RNA functionality.

**Supplementary Information:**

The online version contains supplementary material available at 10.1007/s42994-025-00248-x.

## Introduction

In the epigenomic landscape, histone modifications play crucial roles in regulating gene expression and chromatin dynamics (Zhang et al. 2015). Each type of histone modification can promote or inhibit transcription, depending on the specific context and combination of marks present (Fischle et al. 2015). For instance, the ubiquitination of lysine 120 on histone H2B (H2BK120u1) serves as a mark of actively transcribed genes and promotes the trimethylation of lysines 4 and 79 of histone H3 (H3K4me3 and H3K79me3, respectively) (Guan et al. 2013; Ng et al. 2002; Schulze et al. 2011; Sun and Allis 2002). H3K4me3 promotes histone acetylation (Bian et al. 2011; Clouaire et al. 2012; Crump et al. 2011; Hung et al. 2009; Taverna et al. 2006), but H3K36me2 and H3K36me3 promote histone deacetylation (Carrozza et al. 2005; Joshi and Struhl 2005; Keogh et al. 2005). As repressive marks, H3K27me3 and H2AK119u1 reinforce each other (Blackledge et al. 2014; Cooper et al. 2014; Kalb et al. 2014; Schoeftner et al. 2006; Wang et al. 2004), whereas H3K9me3 and H3K27me3 are typically mutually exclusive (Cooper et al. 2014; Peters et al. 2003; Saksouk et al. 2014). Active marks such as H3K4me2, H3K4me3, H3K36me2, and H3K36me3 inhibit the deposition of H3K27me3 (Ferrari et al. 2014; Gaydos et al. 2012; Schmitges et al. 2011), and the repressive mark H2AK119u1 prevents the methylation of H3K4 and H3K36 (Endoh et al. 2012; Yuan et al. 2013). Despite the usual mutual exclusion of active and repressive histone marks, H3K4me3 and H3K27me3 can coexist on histones covering the promoter regions of a subset of genes in mammalian pluripotent cells (Lesch et al. 2016; Mas et al. 2018). Fifty-one distinct chromatin states can be defined based on the combinations of histone modifications affecting the human genome (Ernst and Kellis 2010). In plants, four main chromatin states can be resolved, based on the combination of 12 chromatin marks (Roudier et al. 2011).

DNA methylation is another critical epigenetic mark that influences chromatin states. Notably, the establishment of DNA methylation is affected by histone modifications. In promoter regions, the extent of H3K4 methylation is negatively correlated with the levels of DNA methylation (Meissner et al. 2008), while DNA methylation shows a positive correlation with H3K36me3 over the gene body (Fu et al. 2020); loss of H3K36me3 leads to depletion of DNA methylation (Xu et al. 2019). In intergenic regions, H3K36me2 is crucial for guiding de novo methylation (Shirane et al. 2020). In constitutive heterochromatin regions, H3K9 methylation reinforces heterochromatin silencing by recruiting DNA methyltransferases and promoting DNA methylation (Dong et al. 2008; Leung et al. 2011; Xin et al. 2003). In contrast, in facultative heterochromatin regions, H3K27 methylation typically antagonizes DNA methylation (Fu et al. 2020; van Mierlo et al. 2019). A strong positive/negative correlation exists between the genome-wide distribution of DNA and histone methylation, highlighting how the interplay of these modifications is fundamental to the regulation of chromatin states.

Beyond DNA and histone modifications, RNA itself carries regulatory information through chemical modifications, forming an additional layer that functionally interacts with the epigenetic landscape. RNA modifications define an epitranscriptomic code and include a wide range of chemical alterations, such as methylation, pseudouridination, and adenosine-to-inosine editing, to name a few (Roundtree et al. 2017). These modifications have been identified in various types of RNA, including ribosomal RNA (rRNA), transfer RNA (tRNA), small nuclear RNA (snRNA), and messenger RNA (mRNA) (Frye et al. 2018). Recently, *N*^*6*^-methyladenosine (m^6^A), the most prevalent internal mRNA modification, was shown to influence chromatin states. Indeed, m^6^A-marked transcripts derived from transposable elements (TEs) recruit an H3K9 methyltransferase, which leads to the deposition of H3K9me3, helping to maintain heterochromatin states and repress transcription of retrotransposons (Chelmicki et al. 2021; Liu et al. 2021; Xu et al. 2021). Histone modifications also influence m^6^A deposition: for instance, H3K36me3 facilitates the binding of the m^6^A methyltransferase complex to RNA polymerase II, promoting the deposition of m^6^A on actively transcribed nascent RNAs (Huang et al. 2019). Furthermore, m^6^A influences DNA methylation, as the m^6^A methyltransferase complex recruits DNA methyltransferases to chromatin for DNA methylation deposition (Kang et al. 2024). Together, DNA methylation and m^6^A—promoting transcription and destabilizing transcripts, respectively—work in concert to regulate the expression of key differentiation genes, thereby influencing embryonic stem cell differentiation in animals (Kang et al. 2024; Liu et al. 2020). mRNA modifications affect the stability, localization, translation, and overall function of mRNAs. Despite studies that have explored the interplay among chromatin modifications, DNA methylation, and mRNA modification, the combined effects of these modifications on mRNA fate remain unclear. In plants, the RNA modifications m^6^A, *N*^*4*^-acetylcytidine (ac^4^C), and 5-methylcytosine (m^5^C) have all been detected on mRNAs, regulating their function (Li et al. 2023; Shao et al. 2021; Shen et al. 2019; Xue et al. 2025); however, the interplay among these modifications is still not fully understood.

In this study, we assessed the co-occurrence of the RNA modifications ac^4^C, m^6^A, and m^5^C on transcripts from Arabidopsis (*Arabidopsis thaliana*) and rice (*Oryza sativa*), using publicly available datasets. We determined that ac^4^C, m^6^A, and m^5^C co-exist on a subset of transcripts. The m^6^A and ac^4^C modifications both inhibited the formation of RNA secondary structures, with m^6^A enhancing the inhibitory effect imposed by ac^4^C. Additionally, the combination of m^6^A and ac^4^C promoted RNA stability. Furthermore, m^6^A enhanced the positive regulation of RNA translational efficiency by ac^4^C.

## Results

### Overlap of plant mRNA modifications

**Fig. 1:**
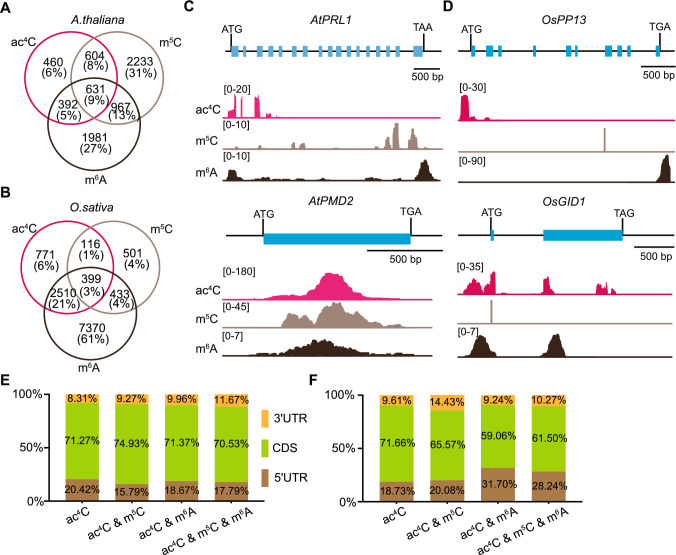
Distribution and overlap of different RNA modifications. **A**, **B** Venn diagrams showing the extent of overlap of transcripts modified with m^6^A, ac^4^C, and/or m^5^C in Arabidopsis (**A**) and rice (**B**). **C** Integrative genomics viewer window of acRIP-seq, m^5^C-seq, and m^6^A-seq reads at the *AtPRL1* and *AtPMD2* loci in Arabidopsis. The *y* axis represents input-subtracted counts per million (CPM). **D** Integrative genomics viewer window of acRIP-seq, m^5^C Bis-seq, and m^6^A-seq reads at the *OsPP13* and *OsGID1* loci in rice. The *y* axis represent input-subtracted CPMs. **E**, **F** Percentages of acRIP-seq peaks in the 5' untranslated region (5' UTR), the coding sequence (CDS), or the 3' UTR of transcripts with different modifications in Arabidopsis (**E**) and rice (**F**)

To investigate the co-occurrence of different mRNA modifications in plants, we reanalyzed published datasets to examine the distribution of the ac^4^C, m^6^A, and m^5^C modifications (Cui et al. 2017; Li et al. 2023; Shen et al. 2016; Tang et al. 2020; Yu et al. 2021). These datasets were derived from similar plant tissues collected at comparable developmental stages and were of similar sequencing depth (Fig. S1A, B). We determined the number of genes whose transcripts were modified with ac^4^C, m^6^A, and/or m^5^C modifications. In Arabidopsis, the transcripts of 1,235 genes exhibited both ac^4^C and m^5^C modifications, while those of 1,023 genes contained ac^4^C and m^6^A, and those of 1,598 genes had both m^6^A and m^5^C, although we can not tell whether the same transcript has both modifications at once. Additionally, the transcripts of 631 genes carried all three modifications (Fig. 1A). In rice, the transcripts of 515 genes showed both ac^4^C and m^5^C modifications, while the transcripts of 2,909 genes contained both ac^4^C and m^6^A, and the transcripts of 832 genes had m^6^A and m^5^C. Furthermore, the transcripts of 399 genes harbored all three modifications (Fig. 1B).

For example, transcripts of *PLEIOTROPIC REGULATORY LOCUS 1* (*AtPRL1*) and *PEROXISOMAL AND MITOCHONDRIAL DIVISION FACTOR 2* (*AtPMD2*) were modified by all three modifications in Arabidopsis, with modifications occurring in different regions on *AtPRL1* transcripts, but overlapping spatially along *AtPMD2* transcripts (Fig. 1C). We observed a similar trend of overlapping modifications for the transcripts of the genes *OsPP13* and *OsGID1* (Fig. 1D). To validate the co-existence of different modifications on the same transcripts, we experimentally assessed the co-existence of ac^4^C and m^6^A on the transcripts of the Arabidopsis genes *AUXIN F-BOX PROTEIN 5* (*AFB5*), *KIN17*, and *LESION INITIATION 2* (*LIN2*) and the rice genes *ALLENE OXIDE SYNTHASE 1* (*AOS1*), *HYBRID PROLINE-RICH PROTEIN 5* (*HyPRP5*), and *STRESS-RESPONSIVE NAC 1* (*SNAC1*), which carried both marks in the above analysis (Fig. S1C, D), using RNA immunoprecipitation followed by reverse-transcription quantitative PCR (Re-RIP-RT-qPCR). We detected significantly higher enrichment of the transcripts from these genes than those of control genes harboring no or only one of the two modifications (Fig. S1E, F), confirming that our analysis accurately detected the co-existence of these RNA modifications.

In Arabidopsis, the ac^4^C modification was enriched near the start and stop codons and within the coding sequence (Fig. S2A). In rice, the distribution of ac^4^C was similar to that in Arabidopsis but exhibited a stronger bias towards the 5' end of transcripts (Fig. S2B). The m^6^A modification was abundant around the stop codon of transcripts in both Arabidopsis and rice, with a notable preference for the 3' end of transcripts in rice (Fig. S2A, B). The m^5^C modification was primarily enriched over the coding sequence; in rice, however, there was also a higher concentration of m^5^C around the start codon (Fig. S2A, B).

The ac^4^C modification has recently been identified as a previously undescribed mRNA modification. To investigate the influence of ac^4^C distribution in relation to other RNA modifications, we analyzed the distribution of ac^4^C in transcripts featuring various modifications. In Arabidopsis, the ac^4^C modification exhibited a different distribution between transcripts with only this modification and those with ac^4^C and other modifications. Specifically, the ac^4^C peaks showed a bias towards the 3' end of transcripts decorated with ac^4^C and m^5^C, as well as those with ac^4^C and m^6^A or all three modifications (Figs. 1E and S2C). In contrast, in rice, the ac^4^C peaks displayed a strong bias toward the 5' end of transcripts with ac^4^C and m^6^A or with all three modifications (Figs. 1F and S2D). Notably, the peaks for modifications on transcripts containing both ac^4^C and m^5^C tended to favor the 3' end of transcripts in rice (Figs. 1F and S2D).

**Fig. 2:**
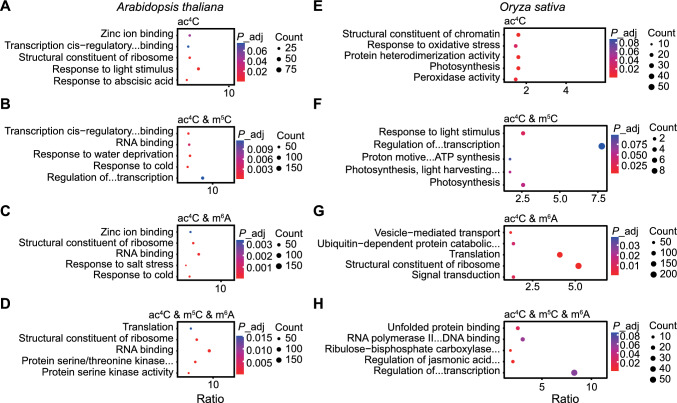
Enriched gene ontology terms enriched in genes containing different RNA modifications. **A**–**D** Enriched gene ontology (GO) terms (biological process and molecular function) for genes whose transcripts are modified by ac^4^C only (**A**), ac^4^C and m^5^C (**B**), ac^4^C and m^6^A (**C**), or ac^4^C and m^5^C and m^6^A (**D**) in Arabidopsis. **E**–**H** Enriched GO terms (biological process and molecular function) for genes whose transcripts are modified by ac^4^C only (**E**), ac^4^C and m^5^C (**F**), ac^4^C and m^6^A (**G**), or ac^4^C and m^5^C and m^6^A (**H**) in rice

Through a gene ontology (GO) analysis, we observed significant enrichment for the genes whose transcripts were co-modified in biological processes related to stress responses in Arabidopsis. For instance, genes whose transcripts were co-modified by ac^4^C and m^5^C were associated with ‘response to cold’, and those whose transcripts were co-modified by ac^4^C and m^6^A were associated with ‘response to salinity stress’ and ‘response to cold’ (Fig. 2A–D). In rice, the genes with transcripts co-modified by ac^4^C and m^5^C were enriched in photosynthesis and translation-related processes (Fig. 2E–H). We also observed enrichment of genes whose transcripts were modified with all three modifications in functions related to protein synthesis, such as ribosome function and amino acid metabolism (Fig. S2E, F). We further categorized transcripts with multiple modifications based on the distance between their peaks: transcripts with overlapping RNA modification peaks were classified in Group I, while those with separate peaks were classified in Group II. In Arabidopsis, Group I genes whose transcripts were co-modified by ac^4^C and m^5^C, as well as genes whose transcripts were co-modified by ac^4^C and m^6^A, were enriched in GO terms related to stress response, while Group II genes for the same modification pairs were enriched in GO terms related to translation and protein binding (Fig. S3A–D). In rice, Group I genes whose transcripts were co-modified by ac^4^C and m^6^A, along with genes whose transcripts were co-modified by all three modifications, were enriched in GO terms related to water deprivation response and translation, while Group II genes for these sets were enriched in GO terms related to tricarboxylic acid cycle and phytohormone response (Fig. S3E–H).

### mRNA modifications collectively influence RNA secondary structure

**Fig. 3:**
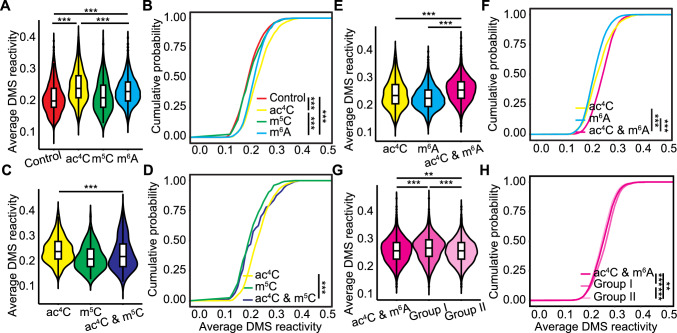
Effects of RNA modifications on RNA secondary structure in rice.** A** Average dimethyl sulfate (DMS) reactivity of control, ac^4^C-modified, m^5^C-modified, or m^6^A-modified transcripts. ***P < 0.001, Kolmogorov–Smirnov test. **B** Cumulative density function plot of the average DMS reactivity of control, ac^4^C-modified, m^5^C-modified, or m^6^A-modified transcripts. ***P < 0.001, Kolmogorov–Smirnov test. **C** Average DMS reactivity of transcripts with ac^4^C only, m^5^C only, or both ac^4^C and m^5^C modifications. ***P < 0.001, Kolmogorov–Smirnov test. **D** Cumulative density function plot of the average DMS reactivity of transcripts with ac^4^C only, m^5^C only, or both ac^4^C and m^5^C modifications. *** *P* < 0.001, Kolmogorov–Smirnov test. **E** Average DMS reactivity of transcripts with ac^4^C only, m^6^A only, or both ac^4^C and m^6^A modifications. ***P < 0.001, Kolmogorov–Smirnov test. **F** Cumulative density function plot of the average DMS reactivity of transcripts with ac^4^C only, m^6^A only, or both ac^4^C and m^6^A modifications. ***P < 0.001, Kolmogorov–Smirnov test. **G** Average DMS reactivity of transcripts with both ac^4^C and m^6^A modifications. The transcripts with both ac^4^C and m^6^A modifications were categorized into two groups. **P < 0.01, ***P < 0.001, Kolmogorov–Smirnov test. **H** Cumulative density function plot of the average DMS reactivity of transcripts with both ac^4^C and m^6^A modifications. **P < 0.01, ***P < 0.001, Kolmogorov–Smirnov test. For all the violin plots, vertical lines indicate the interquartile range and medians are indicated by a black horizontal line

The ac^4^C RNA modification was reported to be negatively correlated with RNA secondary structure in Arabidopsis and rice (Li et al. 2023). Dimethyl sulfate (DMS) can methylate adenines and cytosines in single-stranded regions, potentially leading to termination or mutations during reverse transcription. DMS reactivity is also inversely related to RNA structure and is commonly used as an indicator of RNA conformation strength (Ding et al. 2014). We analyzed the impact of the m^6^A and m^5^C modifications on RNA secondary structure by using a rice RNA structurome dataset (Su et al. 2018). The average DMS reactivity of m^6^A-modified transcripts was higher than that of control transcripts lacking the ac^4^C, m^5^C, or m^6^A modifications, but lower than that of ac^4^C-modified transcripts (Fig. 3A, B). In contrast, the average DMS reactivity of m^5^C-modified transcripts was similar to that of control transcripts, suggesting that m^5^C does not influence RNA secondary structure (Fig. 3A, B).

When we compared transcripts with the ac^4^C and/or m^5^C modifications, the average DMS reactivity of transcripts featuring both ac^4^C and m^5^C was higher than that of m^5^C only–modified transcripts but lower than that of ac^4^C only–modified transcripts (Fig. 3C, D). For transcripts containing both ac^4^C and m^6^A, average DMS reactivity was greater than that of either ac^4^C only–modified or m^6^A only–modified transcripts (Fig. 3E, F). The average DMS reactivity of transcripts with both m^5^C and m^6^A was higher than that of m^5^C only–modified transcripts but similar to that of m^6^A only–modified transcripts (Fig. S4A, B). The average DMS reactivity of transcripts containing all three modifications was higher than that of transcripts with either ac^4^C and m^5^C or both m^5^C and m^6^A; however, their reactivity was similar to that of transcripts with ac^4^C and m^6^A (Fig. S4C, D). We further analyzed the average DMS reactivity of transcripts from the group I and group II gene sets. The transcripts of group I genes, containing both ac^4^C and m^6^A, had significantly higher DMS reactivity than those of group II genes (Fig. 3G, H). Thus, m^6^A enhances the negative regulation of RNA secondary structure imposed by ac^4^C, with an even greater enhancement observed when both modifications overlap, suggesting that m^6^A acts as a cofactor for ac^4^C, possibly by cooperatively disrupting base-pairing. When we conducted a similar analysis after sorting modifications based on where they map on transcripts (5' untranslated region [5' UTR], 3' UTR, coding sequence [CDS]) in Arabidopsis and rice, we noticed that the co-occurrence of ac^4^C and m^5^C in the CDS correlated with lower DMS reactivity (Fig. S5A, B), while the co-occurrence of ac^4^C and m^6^A in different regions resulted in similar DMS reactivity (Fig. S5C, D).

### Regulation of RNA stability by combinations of mRNA modifications

**Fig. 4:**
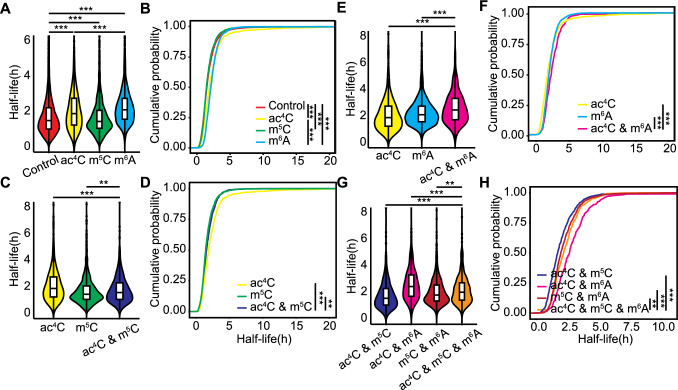
Effects of RNA modifications on RNA stability.** A** Half-lives of control, ac^4^C-modified, m^5^C-modified, or m^6^A-modified transcripts. ***P < 0.001, Kolmogorov–Smirnov test. **B** Cumulative density function plot of the half-lives of control, ac^4^C-modified, m^5^C-modified, or m^6^A-modified transcripts. ***P < 0.001, Kolmogorov–Smirnov test. **C** Half-lives of transcripts with ac^4^C only, m^5^C only, or both ac^4^C and m^5^C modifications. **P < 0.01, ***P < 0.001, Kolmogorov–Smirnov test. **D** Cumulative density function plot of the half-lives of transcripts with ac^4^C only, m^5^C only, or both ac^4^C and m^5^C modifications. **P < 0.01, ***P < 0.001, Kolmogorov–Smirnov test. **E** Half-lives of transcripts with ac^4^C only, m^6^A only, or both ac^4^C and m^6^A modifications. ***P < 0.001, Kolmogorov–Smirnov test. **F** Cumulative density function plot of the half-lives of transcripts with ac^4^C only, m^6^A only, or both ac^4^C and m^6^A modifications. ***P < 0.001, Kolmogorov–Smirnov test. **G** Half-lives of transcripts with ac^4^C and m^5^C, ac^4^C and m^6^A, m^5^C and m^6^A, or all three modifications. **P < 0.01, ***P < 0.001, Kolmogorov–Smirnov test. **H** Cumulative density function plot of the half-lives of transcripts with ac^4^C and m^5^C, ac^4^C and m^6^A, m^5^C and m^6^A, or all three modifications. **P < 0.01, ***P < 0.001, Kolmogorov–Smirnov test

To analyze the effects of mRNA modifications on RNA stability, we assessed the relative stability of transcripts containing ac^4^C, m^6^A, and/or m^5^C using a published dataset (Szabo et al. 2020). In line with existing literature, transcripts modified with ac^4^C and m^6^A demonstrated greater stability than those modified with m^5^C or unmodified transcripts (Fig. 4A, B). Notably, the presence of m^5^C modifications led to a lower relative stability for transcripts also harboring ac^4^C (Fig. 4C, D) or m^6^A (Fig. S6A, B), suggesting that m^5^C may recruit destabilizing proteins such as endonucleases or block access to stabilizing factors bound to m^6^A or ac^4^C. Conversely, the m^6^A modification significantly enhanced the stability of ac^4^C-modified transcripts (Fig. 4E, F). The relative stability of transcripts featuring all three modifications was higher than that of transcripts with both ac^4^C and m^5^C, but lower than that of transcripts with ac^4^C and m^6^A (Fig. 4G, H). Notably, the relative stability of transcripts with all three modifications overlapping was lower than that of transcripts lacking such spatial overlap (Fig. S6C, D), suggesting that the enhancement conferred by m^6^A and ac^4^C is influenced by the spatial arrangement of these modifications. Transcripts with co-occurring ac^4^C and m^5^C modifications in different transcript regions showed similar RNA stability, as did transcripts with co-occurring ac^4^C and m^6^A modifications. This result indicates that the location of these modifications does not affect RNA stability (Fig. S7A–D).

### m^6^A further promotes RNA translation efficiency through ac^4^C

RNA translation efficiency is influenced by RNA modifications. To investigate this question, we measured the translation efficiency of transcripts with various modifications in both Arabidopsis and rice using publicly available datasets (Juntawong et al. 2014; Yang et al. 2020).

**Fig. 5:**
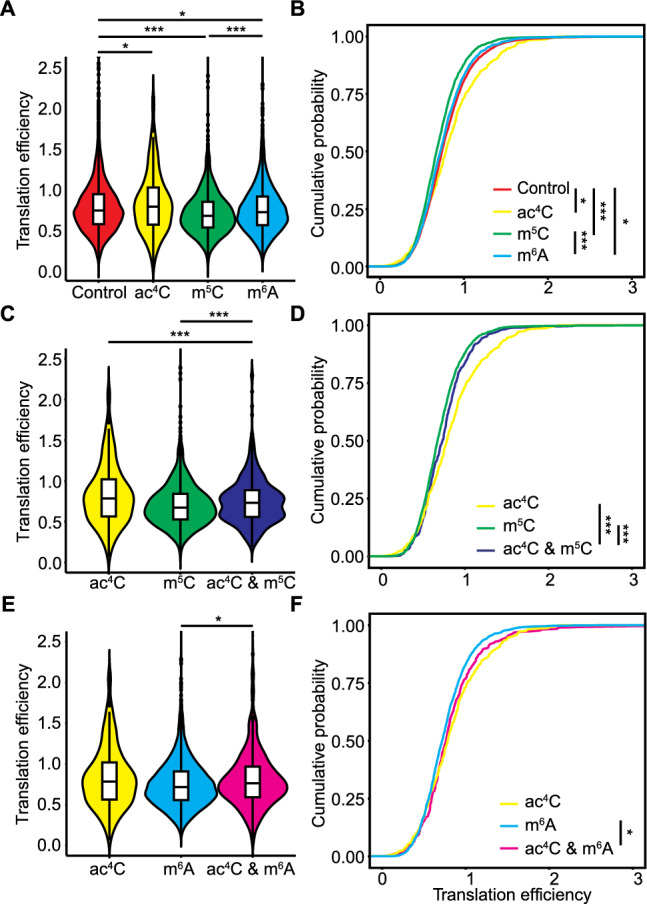
Effects of RNA modifications on RNA translation efficiency in Arabidopsis.** A** Translation efficiency of control, ac^4^C-modified, m^5^C-modified, or m^6^A-modified transcripts. *P < 0.05, ***P < 0.001, Kolmogorov–Smirnov test. **B** Cumulative density function plot of the translation efficiency of control, ac^4^C-modified, m^5^C-modified, or m^6^A-modified transcripts. *P < 0.05, ***P < 0.001, Kolmogorov–Smirnov test. **C** Translation efficiency of transcripts with both ac^4^C and m^5^C modifications. The transcripts with ac^4^C and m^5^C modifications were categorized into two groups. ***P < 0.001, Kolmogorov–Smirnov test. **D** Cumulative density function plot of the translation efficiency of transcripts with both ac^4^C and m^5^C modifications. ***P < 0.001, Kolmogorov–Smirnov test. **E** Translation efficiency of transcripts with both ac^4^C and m^6^A modifications. The transcripts with ac^4^C and m^6^A modifications were categorized into two groups. *P < 0.05, Kolmogorov–Smirnov test. **F** Cumulative density function plot of the translation efficiency of transcripts with both ac^4^C and m^6^A modifications. *P < 0.05, Kolmogorov–Smirnov test

In Arabidopsis, transcripts modified by ac^4^C exhibited higher translation efficiency than control transcripts not modified by ac^4^C, m^6^A, or m^5^C. In contrast, transcripts modified by m^5^C or m^6^A displayed lower translation efficiency than transcripts modified by ac^4^C or not modified (Fig. 5A, B). Transcripts containing both ac^4^C and m^5^C showed a lower translation efficiency than those modified solely by ac^4^C (Fig. 5C, D), while transcripts with ac^4^C and m^6^A exhibited slightly lower translation efficiency than transcripts modified only by ac^4^C (Fig. 5E, F). Notably, transcripts from group I genes modified by both ac^4^C and m^5^C exhibited slightly lower translation efficiency than those from group II genes (Fig. S8A, B), whereas transcripts from group I genes modified by both ac^4^C and m^6^A had a significantly higher translation efficiency than those from group II genes (Fig. S8C, D). This result suggests that m^6^A enhances the translation efficiency promoted by ac^4^C in a spatially dependent manner in Arabidopsis. Transcripts containing m^5^C and m^6^A demonstrated a translation efficiency similar to that of transcripts modified solely by m^5^C or m^6^A (Fig. S8E, F). The translation efficiency of transcripts containing all three modifications was higher than that of transcripts with both m^5^C and m^6^A (Fig. S8G, H). Transcripts with co-occurring ac^4^C and m^5^C modifications showed similar RNA translation efficiency regardless of the distribution of modification peaks along the transcript, as did transcripts with co-occurring ac^4^C and m^6^A modifications (Fig. S9A–D). This finding indicates that the location of these modifications does not affect RNA translation efficiency in Arabidopsis.

**Fig. 6:**
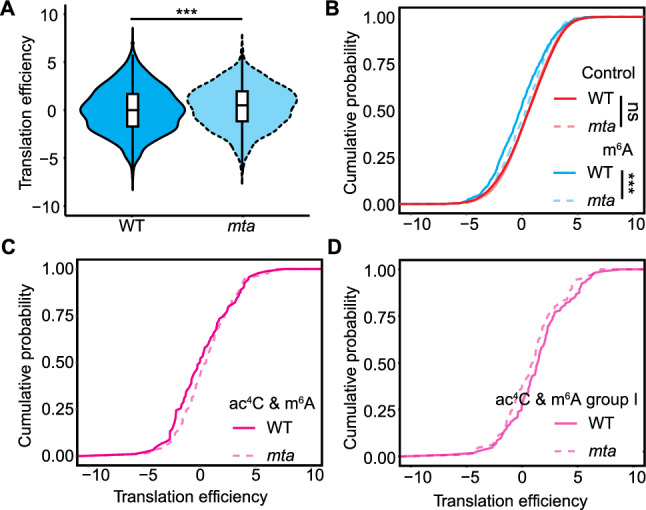
Effects of RNA modifications on RNA translation efficiency in the Arabidopsis *mta* mutant.** A** Translation efficiency of m^6^A-modified transcripts in the wild type (WT, Col-0) or the *mta* mutant. ***P < 0.001, Kolmogorov–Smirnov test. **B** Cumulative density function plot of the translation efficiency of control and m^6^A-modified transcripts in WT or the *mta* mutant. ***P < 0.001, Kolmogorov–Smirnov test. ns, nonsignificant. **C** Translation efficiency of transcripts with both ac^4^C and m^6^A modifications in WT or the *mta* mutant. **D** Cumulative density function plot of the translation efficiency of transcripts with both ac^4^C and m^6^A modifications in WT or the *mta* mutant

To further investigate the role of m^6^A in regulating translation efficiency, we analyzed published datasets (Wang et al. 2023) examining a loss-of-function *MTA* mutant defective in a key m^6^A methyltransferase. Consistent with the known negative correlation between m^6^A and translation efficiency, genes whose transcripts were modified by m^6^A displayed elevated translation efficiency in the *mta* mutant, in which m^6^A deposition is abolished, relative to the wild type (Fig. 6A, B). Genes co-modified with both ac^4^C and m^6^A generally exhibited elevated translation efficiency in *mta* mutant plants (Fig. 6C). However, the transcripts from group I genes showed lower translation efficiency in the wild type than in the *mta* mutant (Fig. 6D). These results support our model whereby m^6^A enhances the translation efficiency promoted by ac^4^C in a spatially dependent manner.

**Fig. 7:**
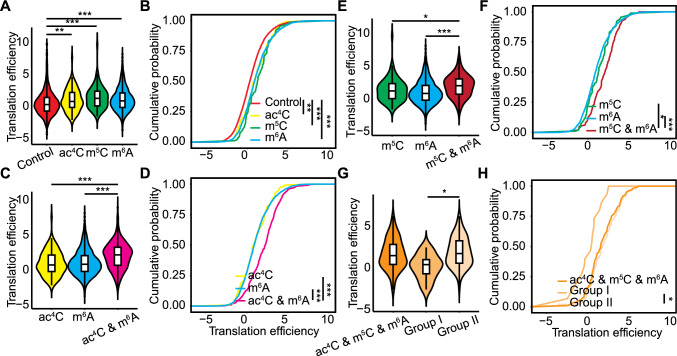
Effects of RNA modifications on RNA translation efficiency in rice.** A** Translation efficiency of control, ac^4^C-modified, m^5^C-modified, and m^6^A-modified transcripts. **P < 0.01, ***P < 0.001, Kolmogorov–Smirnov test. **B** Cumulative density function plot of the translation efficiency of control, ac^4^C-modified, m^5^C-modified, and m^6^A-modified transcripts. **P < 0.01, ***P < 0.001, Kolmogorov–Smirnov test. **C** Translation efficiency of transcripts with ac^4^C only, m^6^A only, or both ac^4^C and m^6^A modifications. ***P < 0.001, Kolmogorov–Smirnov test. **D** Cumulative density function plot of the translation efficiency of transcripts with ac^4^C only, m^6^A only, or both ac^4^C and m^6^A modifications. ***P < 0.001, Kolmogorov–Smirnov test. **E** Translation efficiency of transcripts with m^5^C only, m^6^A only, or both m^5^C and m^6^A modifications. *P < 0.05, ***P < 0.001, Kolmogorov–Smirnov test. **F** Cumulative density function plot of the translation efficiency of transcripts with m^5^C only, m^6^A only, or both m^5^C and m^6^A modifications. *P < 0.05, ***P < 0.001, Kolmogorov–Smirnov test.** G** Translation efficiency of transcripts with ac^4^C, m^5^C, and m^6^A modifications. The transcripts with ac^4^C, m^5^C, and m^6^A modifications were categorized into two groups. *P < 0.05, Kolmogorov–Smirnov test. **H** Cumulative density function plot of the translation efficiency of transcripts with ac^4^C, m^5^C, and m^6^A modifications. *P < 0.05, Kolmogorov–Smirnov test

In rice, all transcripts modified with ac^4^C, m^6^A, or m^5^C exhibited higher translation efficiency than unmodified control transcripts (Fig. 7A, B). Transcripts containing both ac^4^C and m^5^C had a translation efficiency comparable to that of transcripts modified solely by ac^4^C (Fig. S10A, B). In contrast, transcripts with both ac^4^C and m^6^A showed significantly higher translation efficiency than those modified by ac^4^C or m^6^A alone (Fig. 7C, D), and transcripts containing both m^5^C and m^6^A showed higher translation efficiency than those modified solely by m^5^C or m^6^A (Fig. 7E, F). For transcripts with all three modifications, translation efficiency was similar to that of transcripts with the pairs of modifications ac^4^C and m^5^C, ac^4^C and m^6^A, or m^5^C and m^6^A (Fig. S10C, D). Notably, transcripts from group I genes with all three modifications displayed lower translation efficiency than those from group II (Fig. 7G, H), suggesting that these three modifications enhance translation efficiency synergistically in a distance-dependent manner in rice. Transcripts with co-occurring ac^4^C and m^6^A modifications in different transcript regions showed similar translation efficiency in rice, as did those with co-occurring m^5^C and m^6^A modifications. This finding indicates that the location of these modifications does not affect RNA translation efficiency in rice, although their presence does (Fig. S11A–D).

## Discussion

Our findings reveal a complex interplay among the ac^4^C, m^6^A, and m^5^C modifications of plant mRNA, highlighting their combinatorial roles in regulating RNA secondary structure, stability, and translational efficiency. The co-occurrence of these modifications in overlapping regions of transcripts suggests a potential synergistic or antagonistic crosstalk that fine-tunes RNA functionality. Notably, the spatial distribution patterns of these modifications, such as the 5' bias of ac^4^C and m^6^A in rice and their enrichment toward the 3' end of transcripts in Arabidopsis, may reflect species-specific regulatory strategies. The discovery that m^6^A enhances the destabilization of RNA secondary structure caused by deposition of ac^4^C, particularly when the two modifications are spatially clustered, underscores the importance of positional context in the effects of RNA modifications. Furthermore, the observation that m^6^A amplifies ac^4^C-mediated translational efficiency in a distance-dependent manner in Arabidopsis, but not in rice, suggests divergent mechanistic frameworks across plant species. Spatial constraints dictate crosstalk outcomes in a species-specific manner, likely due to distinct RNA-binding protein complexes. Moreover, clustering of modifications leads to stronger RNA destabilization and higher translation efficiency in Arabidopsis but lower translation efficiency in rice; thus, physical proximity of these modifications may enable cooperative recruitment of their respective reader proteins or steric interference. These results expand the current understanding of RNA epitranscriptomics by demonstrating that combinatorial RNA modifications act as a dynamic code to regulate RNA metabolism, potentially serving as a layer of post-transcriptional control in plant development and stress responses.

While this study provides critical insights into the interplay of RNA modifications, several questions remain unresolved. First, the datasets analyzed here are derived from bulk RNA-seq data, which may obscure cell-type-specific or condition-specific dynamic modifications. Second, the mechanistic basis for the cooperative effects of ac^4^C and m^6^A—including whether and how these modifications recruit shared or distinct RNA-binding proteins—requires experimental dissection. Third, the functional consequences of these modifications in specific biological contexts, such as during stress or developmental transitions, remain unexplored. Future studies employing single-molecule sequencing, structural biology, and genetic perturbations could elucidate how the spatial proximity of modifications influences RNA–protein interactions and cellular outcomes. Finally, extending this work to non-model plants and integrating it with chromatin and DNA methylation data may reveal conserved or divergent principles governing the crosstalk between RNA modifications and broader epigenetic and epitranscriptomics networks. Addressing these gaps will deepen our understanding of the RNA modification "code" and its role in plant biology.

## Materials and Methods

### Data accessibility and analysis of sequencing data

All the sequencing data used in this study were publicly available and downloaded from NCBI under the following accession numbers: m^6^A-seq (GEO: GSE135549, GSE7550), acRIP-seq (GEO: GSE198286), and RNA bisulfite sequencing (RNA-BisSeq) (GEO: GSE94065, NGDC: CRA001149) for peak calling and annotation; RNA secondary structure (GEO: GSE100714), RNA stability (GEO: GSE118462), and translation efficiency (GEO: GSE50597, SRA: PRJNA523300); for comparison of translation efficiency in wild-type and *mta* mutant plants, GEO: GSE206292.

Adapters were removed from raw reads with the AdapterRemoval tool (Schubert et al. 2016). The resulting clean reads were mapped to the Arabidopsis (TAIR10) or rice (IRGSP-1.0) reference genome with STAR (v2.5.3a) (Dobin et al. 2013). The R package MeRIPtools (Zhang et al. 2020) was used for m^6^A and ac^4^C peak calling with the following settings: “min_counts = 1, peak_cutoff_fdr = 0.001, peak_cutoff_oddRatio = 1”. The identification of m^5^C methylation sites was performed with the meRnaCall function from the meRanTK tool with the parameter: “-mBQ 20 –mr 0”.

### Computational analysis

Peak annotation was performed with the R package ChIPseeker (Yu et al. 2015). The distance between peaks was calculated using the midpoints of the peaks. Different RNA modification peaks with their midpoints within a 200-bp region were defined as co-occurring within one peak region (group I). Violin plot and cumulative distribution plots were drawn with the R package ggplot2.

Statistical significance was assessed using non-parametric tests. Differences between groups were assessed using the Kolmogorov–Smirnov (KS) test. *P* values were adjusted for multiple comparisons using the Benjamini and Hochberg method.

Only modification sites stably detected across all biological replicate samples were retained. Analyses of transcript stability, dimethyl sulfate (DMS) structural detection, and translation efficiency were uniformly derived from the public available standardized files.

### Re-RIP-RT-qPCR

Total RNAs were fragmented using fragment buffer (NEB) at 94 ºC for 3 min and purified by Trizol. RNAs were heated to 70 ºC for 5 min and transferred to ice for 2 min to denature RNA secondary structure. Denatured RNAs (50 µg) were diluted to 1 mL using RNA immunoprecipitation buffer (RIP buffer) containing 20 mM Tris–HCl (pH 7.9, 4 ºC), 1 mM MgCl_2_, 200 mM KCl, 0.05% IGEPAL CA-630 and 40U murine RNase inhibitor (NEB). 1 µg of ac^4^C antibody (Abcam ab252215) was added into diluted RNAs and incubated at 4 ºC for 4 h. Immunocomplexes were recovered with pre-coated protein G Dynabeads (Thermo). 1% mixture was taken as input. The beads were washed five times with RIP buffer, supernatant was carefully removed after the final wash. Input and immunoprecipitated RNAs were purified by Trizol. The immunoprecipitated RNAs were then diluted in RIP buffer and precipitated by 1 µg of m^6^A antibody (Synaptic Systems, 202,003).

For quantitative reverse transcription-polymerase chain reaction (qRT-PCR), reverse transcription was performed using SuperScript™ IV Reverse Transcriptase (Thermo Fisher 18,090,010) according to the manufacturer’s instructions. Three biological replicates were analyzed and each was tested by three technical replicates. Real-time PCR was performed using gene-specific primers and the results were normalized against input samples to represent the relative enrichment.

## Supplementary Information

Below is the link to the electronic supplementary material.

Supplementary file1 (DOCX 1811 KB)

## Data Availability

All the sequencing data used in this study were publicly available and were downloaded from NCBI under the following accession numbers: m6A-seq (GEO: GSE135549, GSE7550), acRIP-seq (GEO: GSE198286), and RNA bisulfite sequencing (RNA-BisSeq) (GEO: GSE94065, NGDC: CRA001149) for peak calling and annotation; RNA secondary structure (GEO: GSE100714), RNA stability (GEO: GSE118462), and translation efficiency (GEO: GSE50597, SRA: PRJNA523300); for comparison of translation efficiency in wild-type and mta mutant plants, GEO: GSE206292.
