# Supplementary material for: Synergistic function of RNA modifications in Arabidopsis and rice
Source: aBIOTECH. 2025 Oct 9;6(4):803–15. doi: 10.1007/s42994-025-00248-x (PMC12647476; doi:10.1007/s42994-025-00248-x)
Supplement: Supplementary file 1 — Supplementary file1 (DOCX 1811 KB) [file 42994_2025_248_MOESM1_ESM.docx]

**Supplemental Information**

**Synergistic function of RNA modifications in plants**

Ancheng Ma^1,2,3^, Shuaibin Wang^1^, Xinxi He^1^, Yongbo Qu^1^, Shenglin Xie^1^, Junpin Gao^1^, Yu Peng^1^, Lisha Shen^4^, Wenxuan Pu^1,*^, Chongsheng He^2,3,*^

^1^Tobacco Research Institute of Technology Centre, China Tobacco Hunan Industrial Corporation, Changsha 410014, China

^2^Hunan Key Laboratory of Plant Functional Genomics and Developmental Regulation, College of Biology, Hunan University, Changsha 410082, China

^3^Hunan Research Center of the Basic Discipline for Cell Signaling

^4^Temasek Life Sciences Laboratory, National University of Singapore, 117604, Singapore

Ancheng Ma, Shuaibin Wang have contributed equally to this work.

^*^Correspondence author: Wenxuan Pu: puwenxuan_2022@163.com; Chongsheng He: chongshenghe@outlook.com.

**Inventory of Supplemental Information**

**Fig. S1** Validation of co-existence of ac^4^C and m^6^A.

**Fig. S2** Distribution of ac^4^C on transcripts with different modifications.

**Fig. S3** Additional analyses of enriched GO terms for genes containing different RNA modifications.

**Fig. S4** Additional analyses of effects of RNA modifications on RNA secondary structure.

**Fig. S5** Additional analyses of effects of RNA modifications within different gene regions on RNA secondary structure.

**Fig. S6** Additional analyses of effects of RNA modifications on RNA stability.

**Fig. S7** Additional analyses of effects of RNA modifications within different gene regions on RNA stability.

**Fig. S8** Additional analyses of effects of RNA modifications on RNA translation efficiency in *Arabidopsis*.

**Fig. S9** Additional analyses of effects of RNA modifications within different gene regions on RNA translation efficiency in *Arabidopsis*.

**Fig. S10** Additional analyses of effects of RNA modifications on RNA translation efficiency in rice.

**Fig. S11** Additional analyses of effects of RNA modifications within different gene regions on RNA translation efficiency in rice.


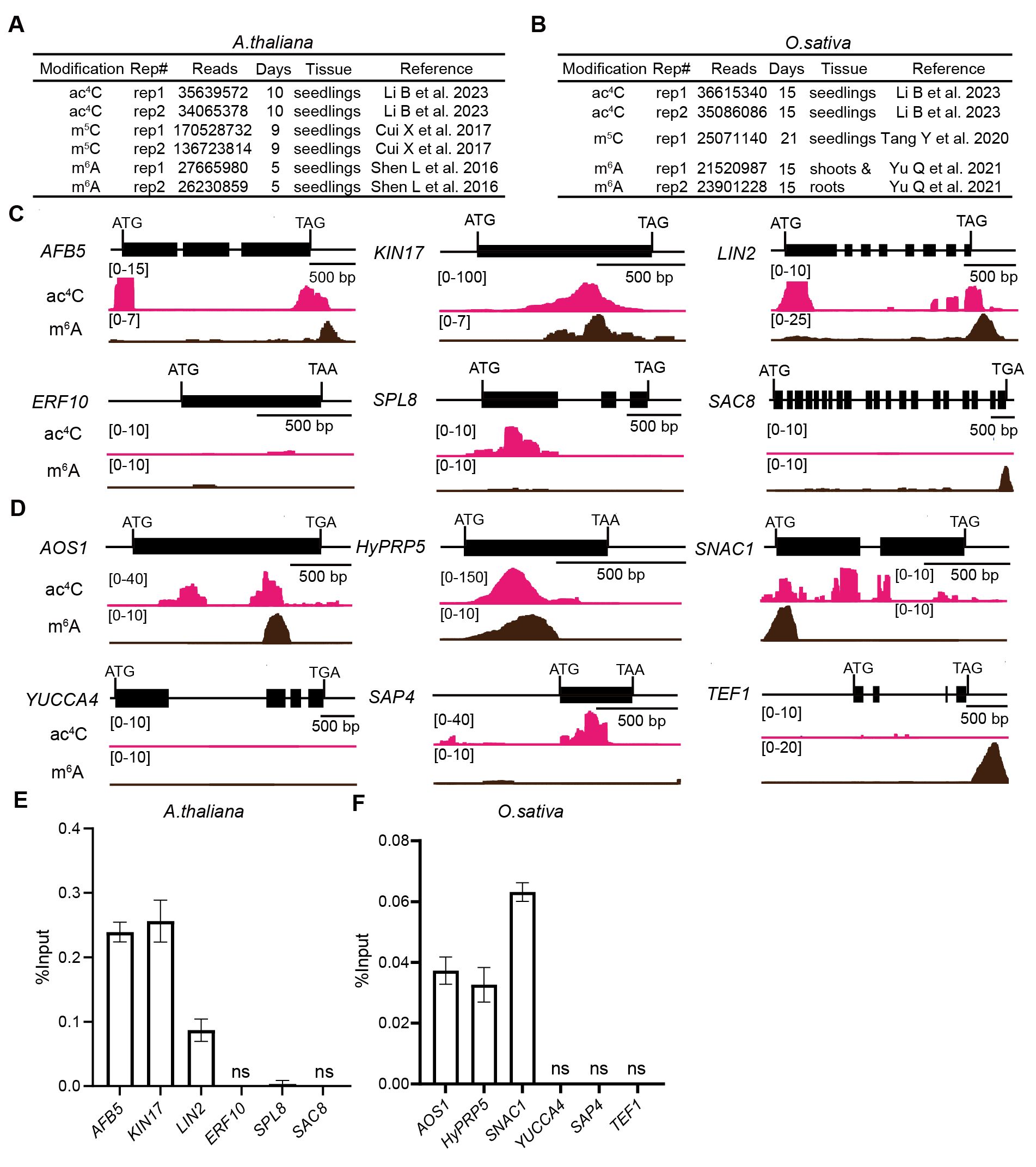


Fig. S1 Validation of co-existence of ac^4^C and m^6^A. A Information of *Arabidopsis* ac^4^C, m^5^C and m^6^A sequencing datasets used in this study. B Information of rice ac^4^C, m^5^C and m^6^A sequencing datasets used in this study. C Genomic browser snapshot of *Arabidopsis* acRIP-seq and m^6^A-seq reads at the *AFB5*, *KIN17*, *LIN2*, *ERF10*, *SPL8* and *SAC8* loci. The *y* axis represented input subtracted CPMs. D Genomic browser snapshot of rice acRIP-seq and m^6^A-seq reads at the *AOS1*, *HYPRP5*, *SNAC1*, *YUCCA4*, *SAP4* and *TEF1* loci. The *y* axis represented input subtracted CPMs. E qPCR results of co-existence of ac^4^C and m^6^A on *AFB5*, *KIN17*, *LIN2*, *ERF10*, *SPL8* and *SAC8* transcripts*.* RNA was first immunoprecipitated and purified using an m^6^A antibody, the resulting m^6^A-enriched RNA was then subjected to ac^4^C-RIP, followed by quantitative PCR analysis. F qPCR results of co-existence of ac^4^C and m^6^A on *AOS1*, *HYPRP5*, *SNAC1*, *YUCCA4*, *SAP4* and *TEF1* transcripts*.* RNA was first immunoprecipitated and purified using an m^6^A antibody, the resulting m^6^A-enriched RNA was then subjected to ac^4^C-RIP, followed by quantitative PCR analysis.


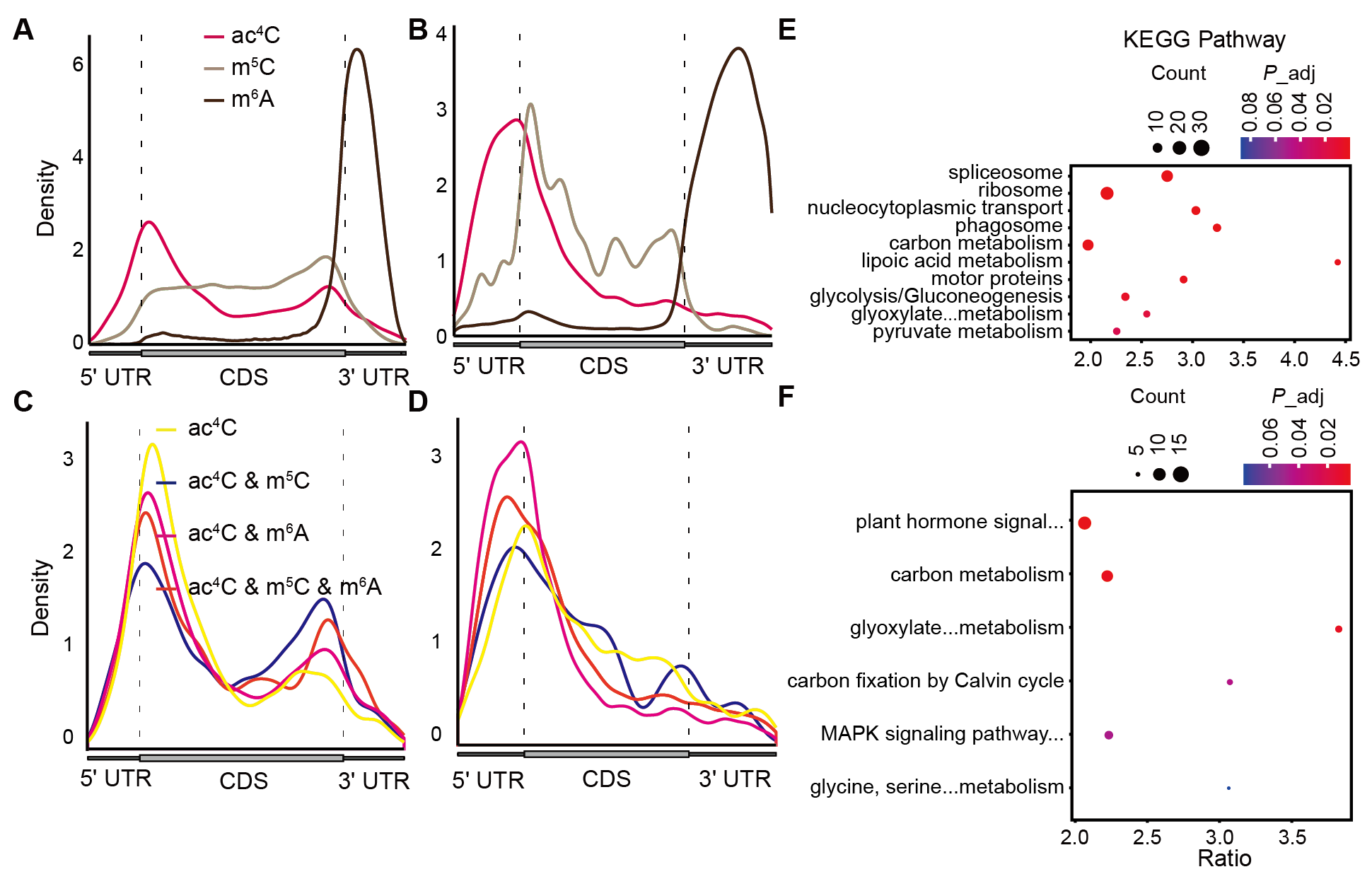


**Fig. S2** Distribution of ac^4^C on transcripts with different modifications. **A** Distribution of m^6^A-seq, acRIP-seq and m5C-seq reads along transcripts in *Arabidopsis*. Each transcript is divided into 5’ UTR, CDS, 3’ UTR. The *y* axis represents the density of reads. **B** Distribution of m^6^A-seq, acRIP-seq and m5C-seq reads along transcripts in rice. **C** Distribution of acRIP-seq reads from transcripts with different modifications along transcripts in *Arabidopsis*. Each transcript is divided into 5’ UTR, CDS, 3’ UTR. The *y* axis represents the density of reads. **D** Distribution of acRIP-seq reads from transcripts with different modifications along transcripts in rice. Each transcript is divided into 5’ UTR, CDS, 3’ UTR. The *y* axis represents the density of reads. **E** Enriched KEGG pathways of genes modified by ac^4^C & m^5^C & m^6^A in *Arabidopsis*. **F** Enriched KEGG pathways of genes modified by ac^4^C & m^5^C & m^6^A in rice.


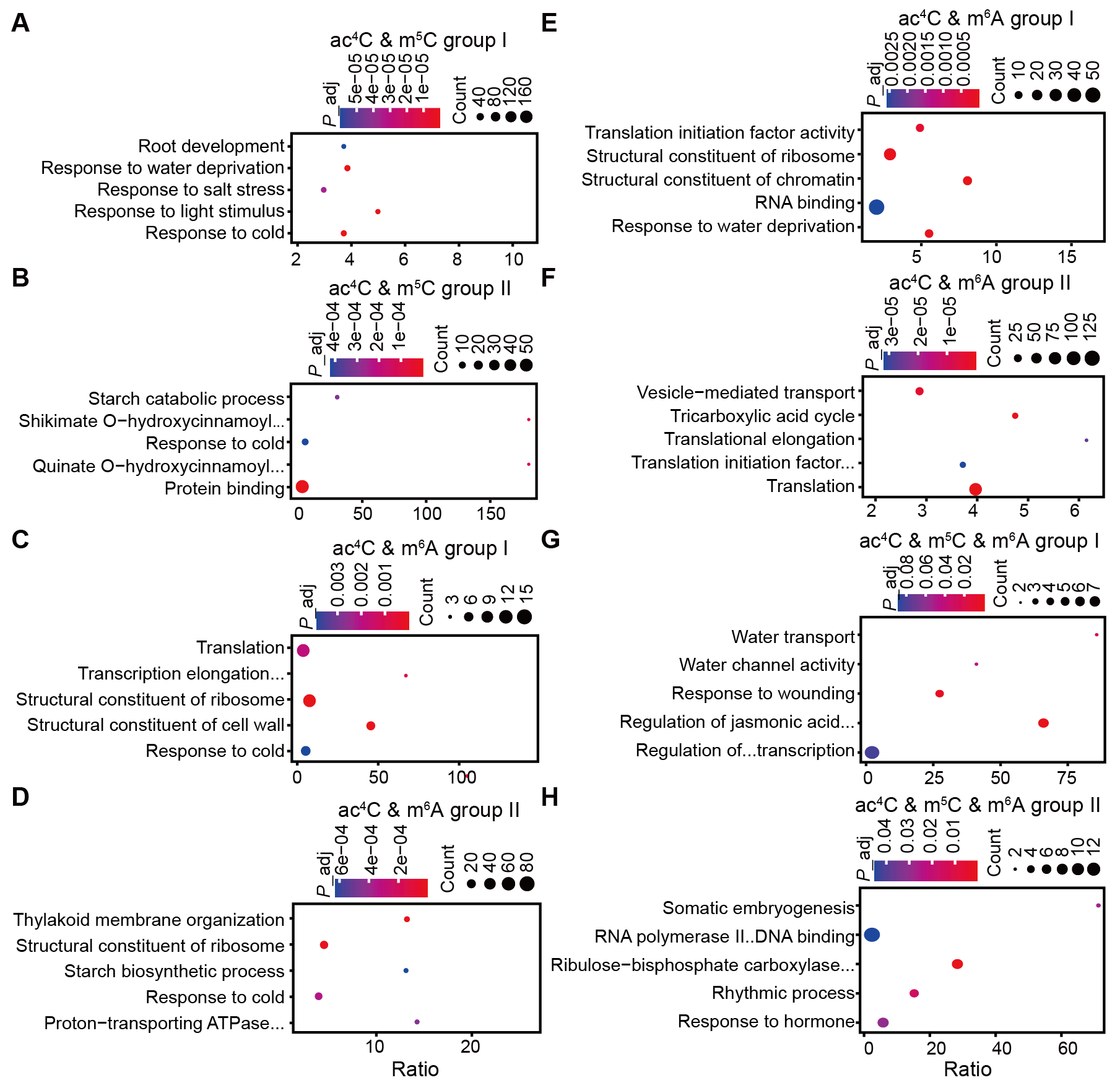


**Fig. S3** Additional analyses of enriched GO terms for genes containing different RNA modifications. **A-D** Enriched GO terms (biological process and molecular function) for genes modified by ac^4^C & m^5^C and ac^4^C & m^6^A in *Arabidopsis*. The transcripts with both ac^4^C & m^5^C and ac^4^C & m^6^A modifications were categorized into two groups. **E-H** Enriched GO terms (biological process and molecular function) for genes modified by ac^4^C & m^6^A and ac^4^C & m^5^C & m^6^A in rice. The transcripts with both ac^4^C & m^6^A and ac^4^C & m^5^C & m^6^A modifications were categorized into two groups.


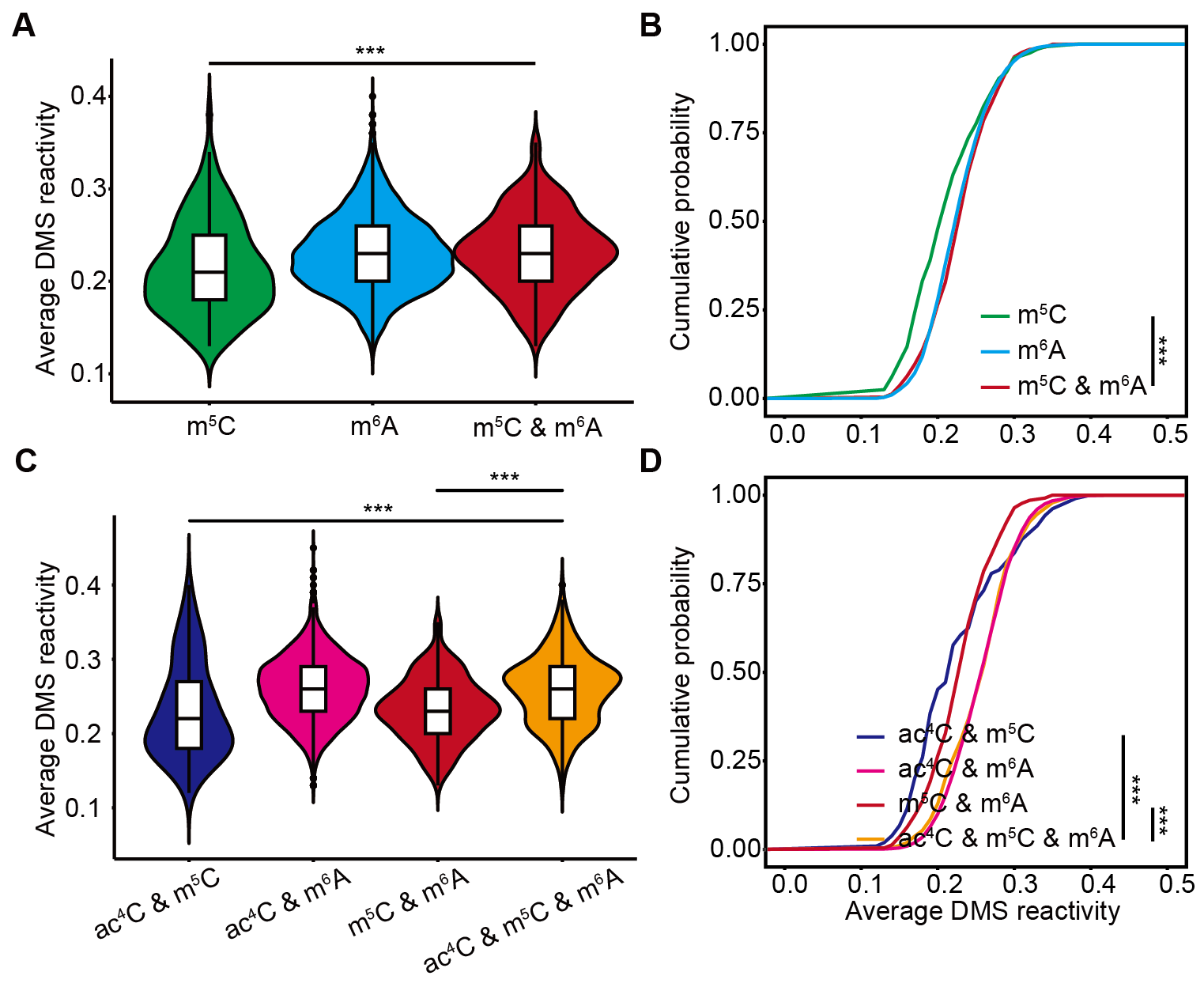


**Fig. S4** Additional analyses of effects of RNA modifications on RNA secondary structure. **A** The average DMS reactivity of transcripts with m^5^C-only, m^6^A-only and both m^5^C and m^6^A modifications. *** *P* < 0.001, Kolmogorov-Smirnov test. **B** CDF plot of the average DMS reactivity of transcripts with m^5^C-only, m^6^A-only and both m^5^C and m^6^A modifications. *** *P* < 0.001, Kolmogorov-Smirnov test. **C** The average DMS reactivity of transcripts with ac^4^C & m^5^C, ac^4^C & m^6^A, m^5^C & m^6^A and all the three modifications. *** *P* < 0.001, Kolmogorov-Smirnov test. **D** CDF plot of the average DMS reactivity of transcripts with ac^4^C and m^5^C, ac^4^C and m^6^A, m^5^C and m^6^A and all the three modifications. *** *P* < 0.001, Kolmogorov-Smirnov test.


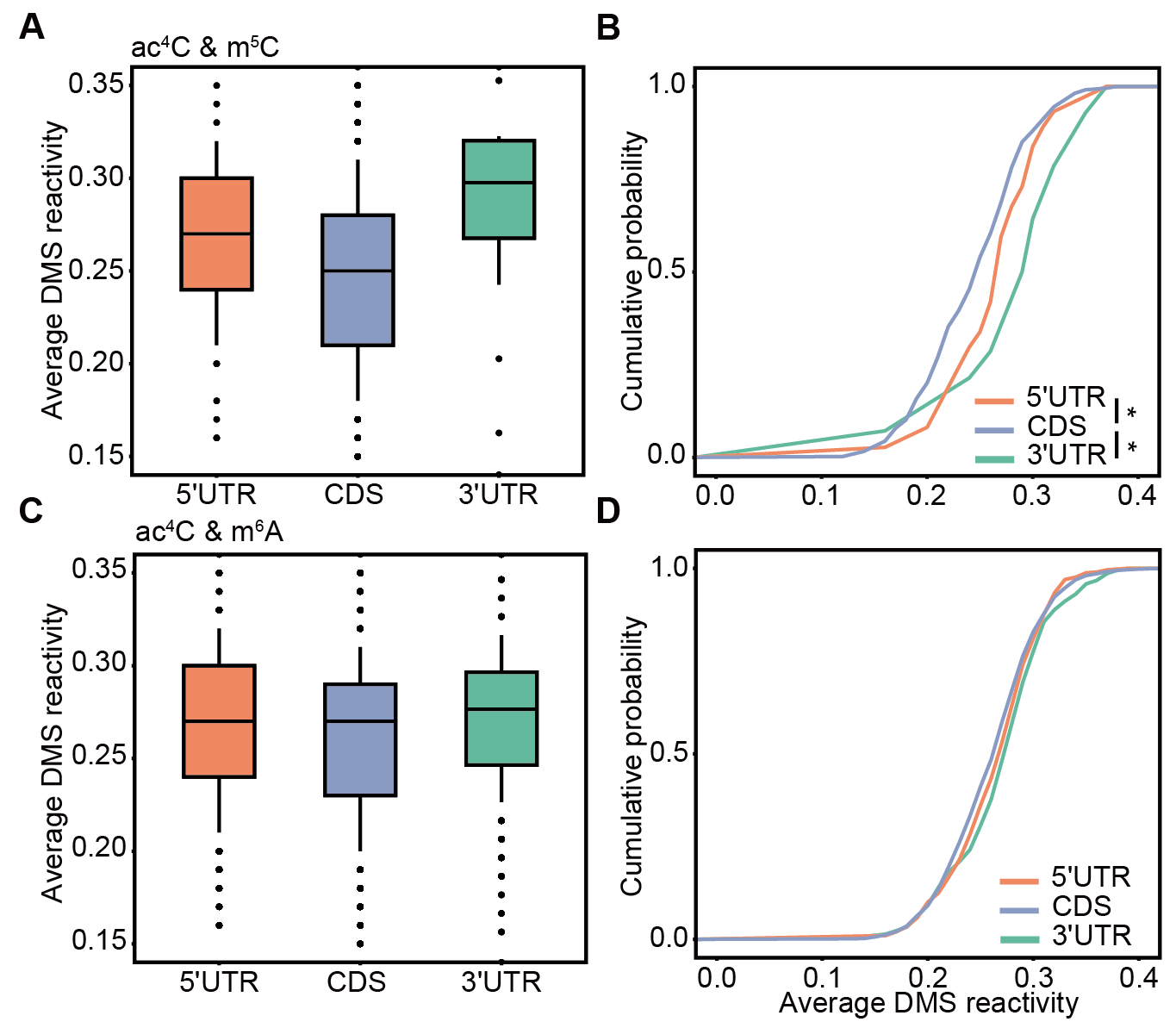


**Fig. S5** Additional analyses of effects of RNA modifications within different gene regions on RNA secondary structure. **A** The average DMS reactivity of transcripts with ac^4^C & m^5^C modifications fall into 5’UTR, CDS and 3’UTR regions. **B** CDF plot of the average DMS reactivity of transcripts with ac^4^C & m^5^C modifications fall into 5’UTR, CDS and 3’UTR regions. * *P* < 0.05, Kolmogorov-Smirnov test. **C** The average DMS reactivity of transcripts with ac^4^C & m^6^A modifications fall into 5’UTR, CDS and 3’UTR regions. **D** CDF plot of the average DMS reactivity of transcripts with ac^4^C & m^6^A modifications fall into 5’UTR, CDS and 3’UTR regions.

**
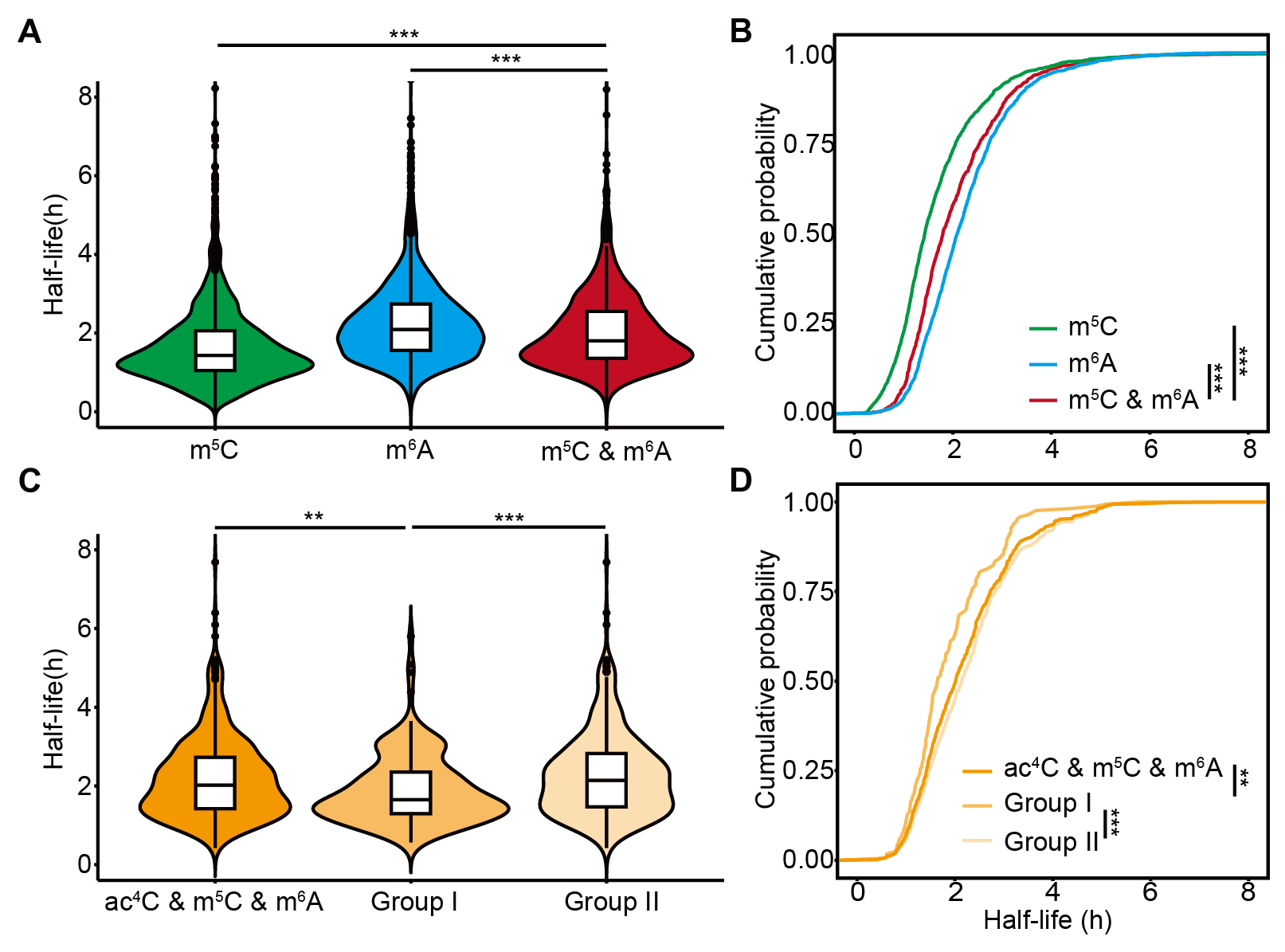
**

**Fig. S6** Additional analyses of effects of RNA modifications on RNA stability. **A** The half-life of transcripts with m^5^C-only, m^6^A-only and both m^5^C and m^6^A modifications. *** *P* < 0.001, Kolmogorov-Smirnov test. **B** CDF plot of the half-life of transcripts with m^5^C-only, m^6^A-only and both m^5^C and m^6^A modifications. *** *P* < 0.001, Kolmogorov-Smirnov test. **C** The half-life of transcripts with all the three modifications. The transcripts with all the three modifications were categorized into two groups. ** *P* < 0.05, *** *P* < 0.001, Kolmogorov-Smirnov test. **D** CDF plot of the half-life of transcripts with all the three modifications. The transcripts with all the three modifications were categorized into two groups. ** *P* < 0.01, *** *P* < 0.001, Kolmogorov-Smirnov test.

**
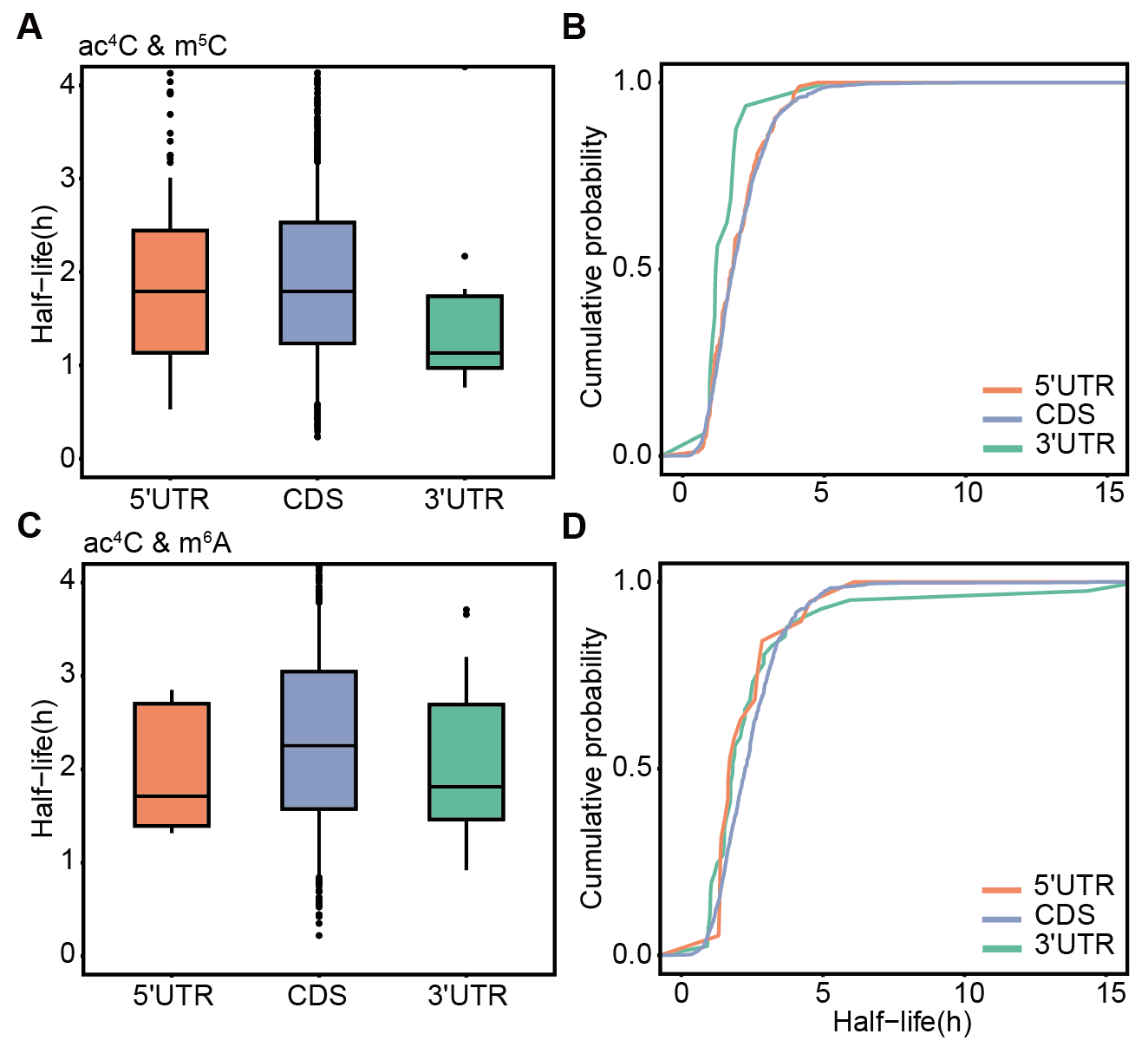
**

**Fig. S7** Additional analyses of effects of RNA modifications within different gene regions on RNA stability. **A** The half-life of transcripts with ac^4^C & m^5^C modifications fall into 5’UTR, CDS and 3’UTR regions. **B** CDF plot of the half-life of transcripts with ac^4^C & m^5^C modifications fall into 5’UTR, CDS and 3’UTR regions. **C** The half-life of transcripts with ac^4^C & m^6^A modifications fall into 5’UTR, CDS and 3’UTR regions. **D** CDF plot of the half-life of transcripts with ac^4^C & m^6^A modifications fall into 5’UTR, CDS and 3’UTR regions.

**
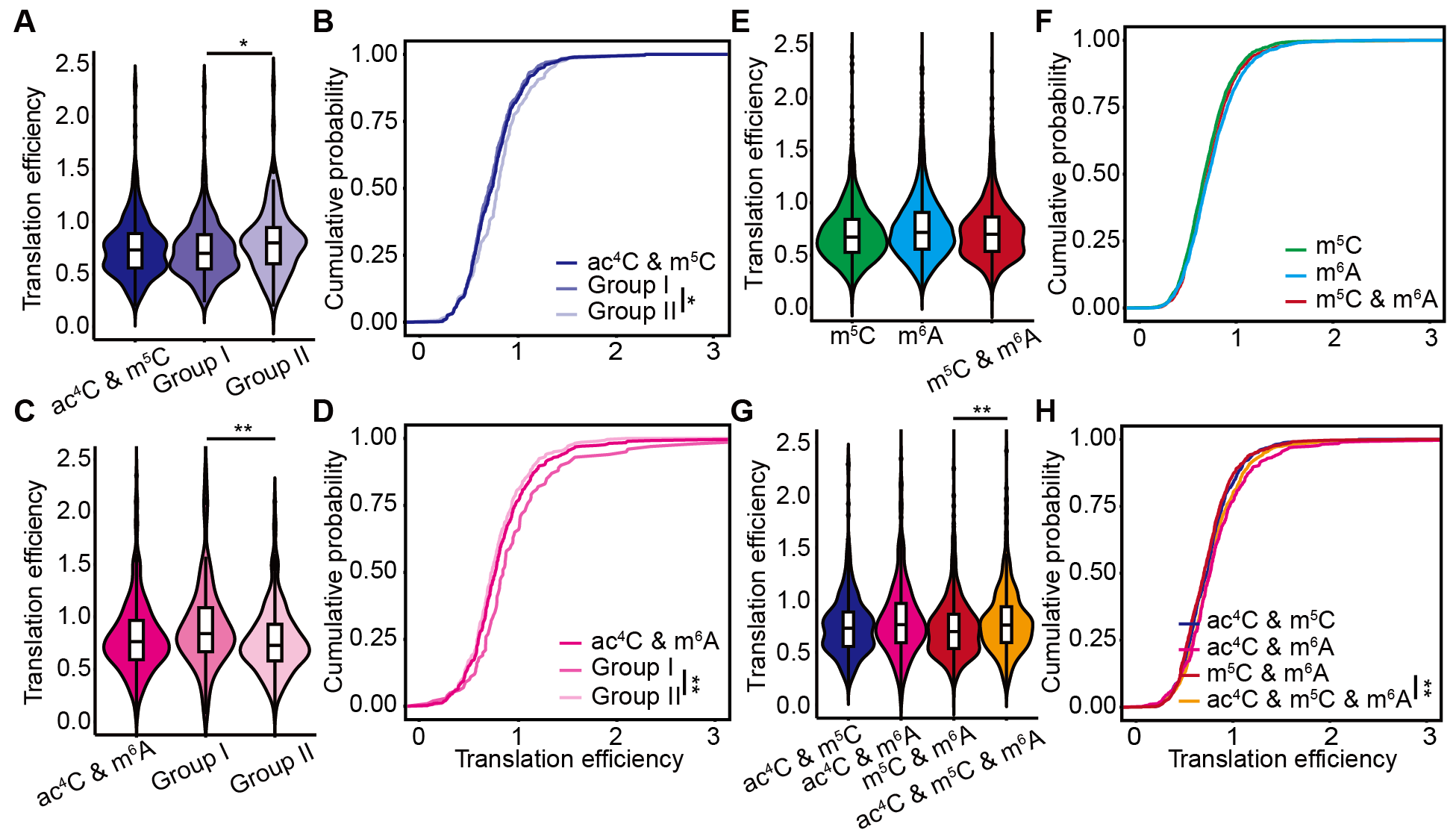
**

**Fig. S8** Additional analyses of effects of RNA modifications on RNA translation efficiency in *Arabidopsis*. **A** The translation efficiency of transcripts with both ac^4^C and m^5^C modifications. The transcripts with both ac^4^C and m^5^C modifications were categorized into two groups. * *P* < 0.05, Kolmogorov-Smirnov test. **B** CDF plot of the translation efficiency of transcripts with both ac^4^C and m^5^C modifications. * *P* < 0.05, Kolmogorov-Smirnov test. **C** The translation efficiency of transcripts with both ac^4^C and m^6^A modifications. The transcripts with both ac^4^C and m^6^A modifications were categorized into two groups. * *P* < 0.05, Kolmogorov-Smirnov test. **D** CDF plot of the translation efficiency of transcripts with both ac^4^C and m^6^A modifications. The transcripts with both ac^4^C and m^6^A modifications were categorized into two groups. * *P* < 0.05, Kolmogorov-Smirnov test. **E** The translation efficiency of transcripts with m^5^C-only, m^6^A-only and both m^5^C and m^6^A modifications. **F** CDF plot of the translation efficiency of transcripts with m^5^C-only, m^6^A-only and both m^5^C and m^6^A modifications. **G** The translation efficiency of transcripts with ac^4^C & m^5^C, ac^4^C & m^6^A, m^5^C & m^6^A and all the three modifications. ** *P* < 0.01, Kolmogorov-Smirnov test. **H** CDF plot of the translation efficiency of transcripts with ac^4^C & m^5^C, ac^4^C & m^6^A, m^5^C & m^6^A and all the three modifications. ** *P* < 0.01, Kolmogorov-Smirnov test.

**
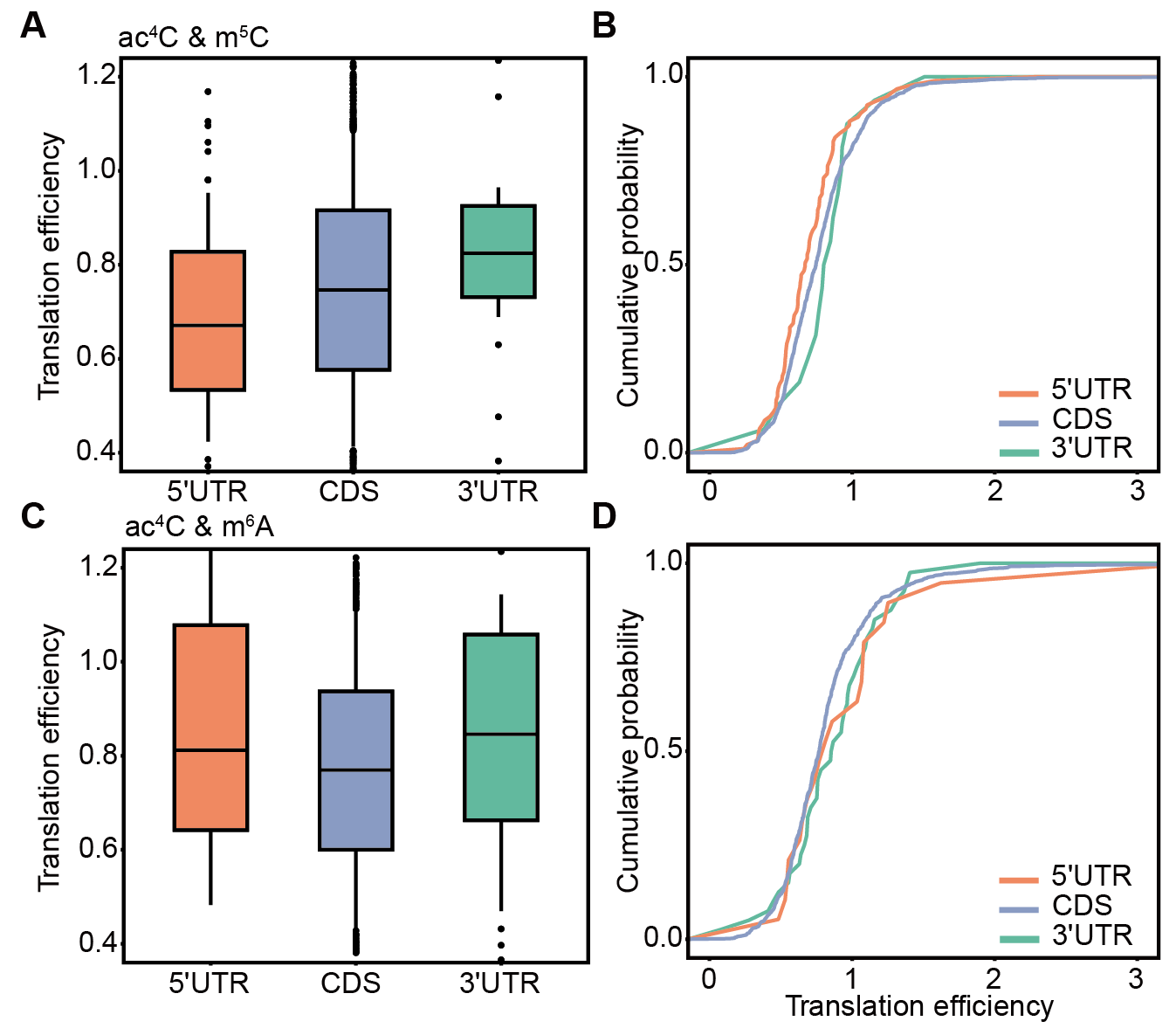
**

**Fig. S9** Additional analyses of effects of RNA modifications within different gene regions on RNA translation efficiency in *Arabidopsis*. **A** The translation efficiency of transcripts with ac^4^C & m^5^C modifications fall into 5’UTR, CDS and 3’UTR regions. **B** CDF plot of the translation efficiency of transcripts with ac^4^C & m^5^C modifications fall into 5’UTR, CDS and 3’UTR regions. **C** The translation efficiency of transcripts with ac^4^C & m^6^A modifications fall into 5’UTR, CDS and 3’UTR regions. **D** CDF plot of the translation efficiency of transcripts with ac^4^C & m^6^A modifications fall into 5’UTR, CDS and 3’UTR regions.

**
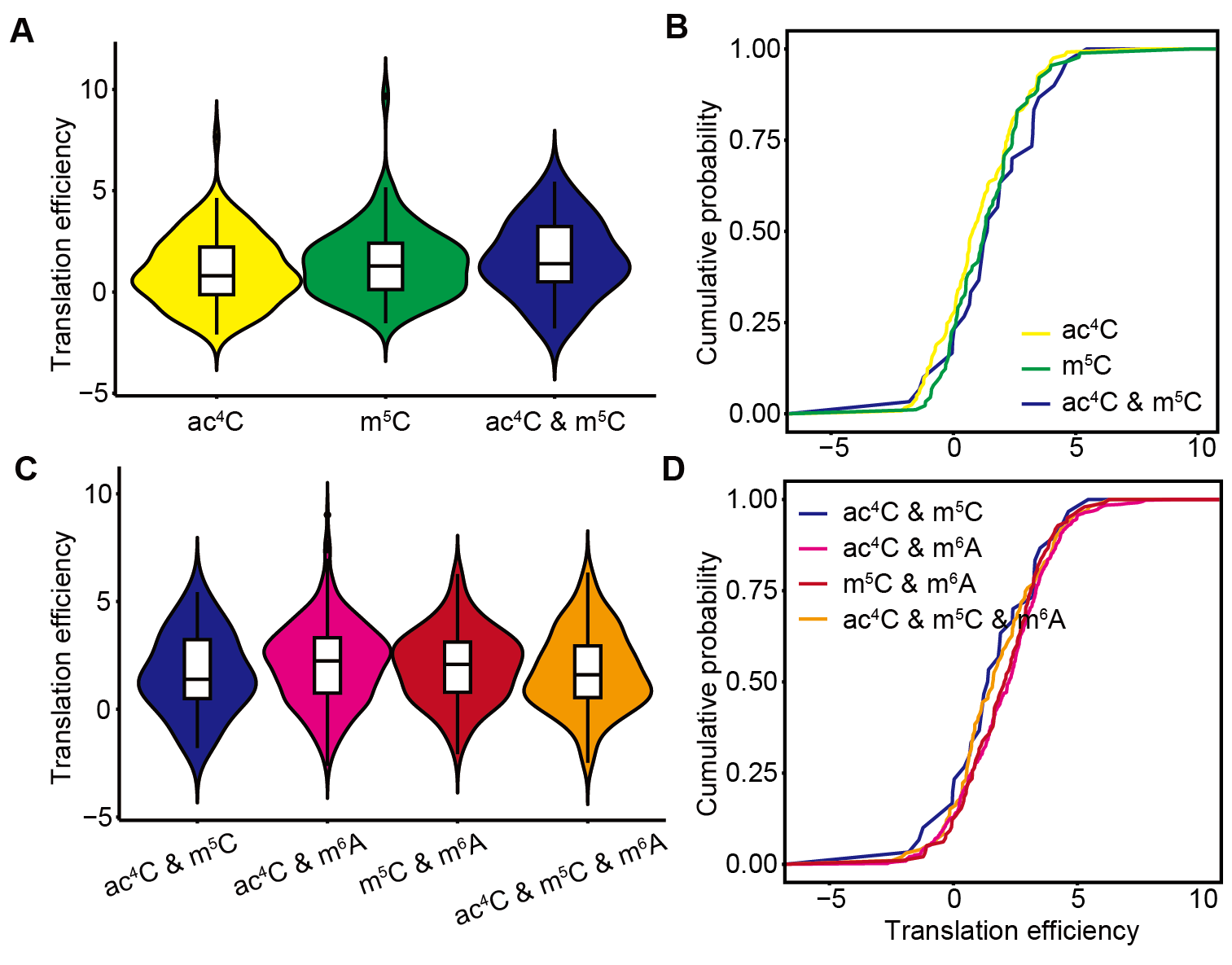
**

**Fig. S10** Additional analyses of effects of RNA modifications on RNA translation efficiency in rice. **A** The translation efficiency of transcripts with ac^4^C-only, m^5^C-only and both ac^4^C & m^5^C modifications. **B** CDF plot of the translation efficiency of transcripts with ac^4^C-only, m^5^C-only and both ac^4^C & m^5^C modifications. **C** The translation efficiency of transcripts with ac^4^C & m^5^C, ac^4^C & m^6^A, m^5^C & m^6^A and all the three modifications. **H** CDF plot of the translation efficiency of transcripts with ac^4^C & m^5^C, ac^4^C & m^6^A, m^5^C & m^6^A and all the three modifications.

**
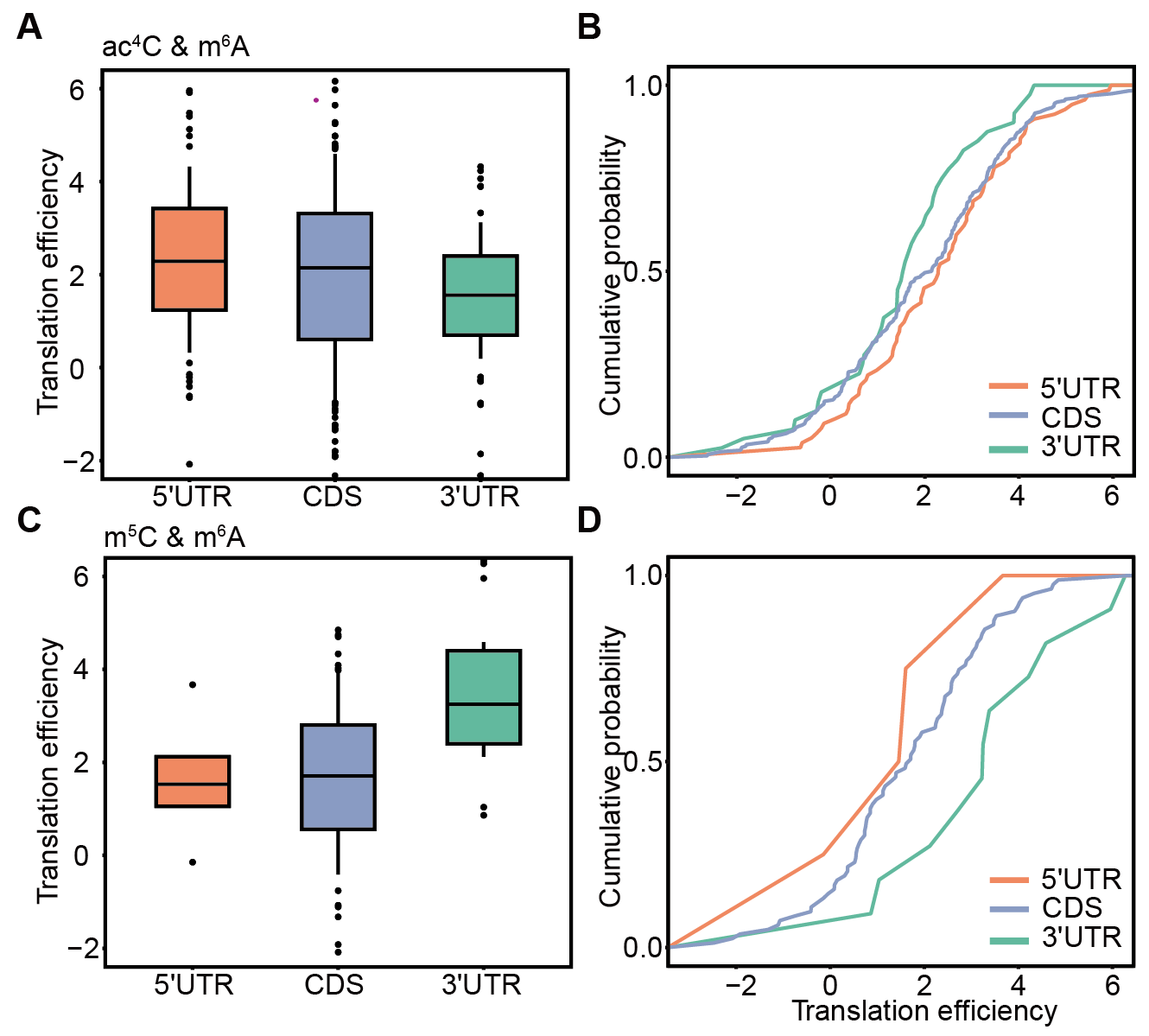
**

**Fig. S11** Additional analyses of effects of RNA modifications within different gene regions on RNA translation efficiency in rice. **A** The translation efficiency of transcripts with ac^4^C & m^6^A modifications fall into 5’UTR, CDS and 3’UTR regions. **B** CDF plot of the translation efficiency of transcripts with ac^4^C & m^6^A modifications fall into 5’UTR, CDS and 3’UTR regions. **C** The translation efficiency of transcripts with m^5^C & m^6^A modifications fall into 5’UTR, CDS and 3’UTR regions. **D** CDF plot of the translation efficiency of transcripts with m^5^C & m^6^A modifications fall into 5’UTR, CDS and 3’UTR regions.
